# Transcranial magnetic stimulation combined with endogenous human hippocampal and motor cortical activity enhances memory

**DOI:** 10.1371/journal.pone.0295413

**Published:** 2023-12-07

**Authors:** Arantzazu San Agustín, David Crevillén, Vanesa Soto-León, Juan C. Moreno, Antonio Oliviero, José L. Pons

**Affiliations:** 1 Neural Rehabilitation Group (NRG), Cajal Institute, Consejo Superior de Investigaciones Científicas (CSIC), Madrid, Spain; 2 PhD Program in Neuroscience, Universidad Autonoma de Madrid-Cajal Institute, Madrid, Spain; 3 Department of Physical Medicine and Rehabilitation, Feinberg School of Medicine, Northwestern University, Chicago, IL, United States of America; 4 Legs & Walking AbilityLab, Shirley Ryan AbilityLab, Chicago, IL, United States of America; 5 FENNSI Group, Hospital Nacional de Parapléjicos, SESCAM, Toledo, Spain; 6 Center for Clinical Neuroscience, Hospital Los Madroños, Brunete, Spain; Universita degli Studi di Torino, ITALY

## Abstract

The hippocampus is a fundamental cortical structure in the memory process of encoding, retaining, and recalling information. Transcranial Magnetic Stimulation (TMS) following a Paired Associative Stimulation (PAS) enhances nervous system excitability and promotes cortical plasticity mechanisms by synchronizing two stimuli in the same neural pathway. However, PAS has not been shown to improve memorization capacity yet. Here, we present an innovative protocol stemming from the PAS paradigm, which combines single-pulse TMS to the hippocampus with endogenous hippocampal activity during a working memory (WM) task. 96 volunteers were randomized across one experimental group and three parallel groups (motor cortex stimulation, sham stimulation, and no stimulation) in a single session. This combined-stimuli configuration resulted in an increased memorization capacity in the WM task, which was dependent on the stimulated brain location and subjects’ basal memory performance. These results are potentially significant for clinical research on memory dysfunction and its related neurological disorders. Future research on paired associative or combined stimulation is required to unveil stimulation-derived neural mechanisms that enhance the ability to memorize.

## Introduction

While the complete understanding of the **cognitive process of memorization** remains unknown, damage to the hippocampus is sufficient to cause easily detectable and clinically significant memory impairment in humans [1, 2]. Severe memory impairment is observed when the lesion includes the para-hippocampal gyrus, entorhinal cortex, or part of the perirhinal cortex [3, 4]. These cortical areas have been commonly associated with long-term episodic memory [5, 6]. However, other studies have also shown visuospatial working memory (WM) disruption with hippocampal damage [7–10].

At the cellular level, synaptic plasticity has been established as the basic mechanism of learning and memory. In 1949, Donald Hebb explained the synaptic dynamic regulation, emphasizing the importance of space and time in which neurons are activated [11]. Starting from this postulate, **Spike-Timing-Dependent Plasticity (STDP)** has been demonstrated as an associative synaptic plasticity form in which the temporal synchronization of presynaptic and postsynaptic activation determines the induction of synaptic potentiation or depression.

Non-invasive brain stimulation (NIBS) techniques such as **Transcranial Magnetic Stimulation (TMS)** have enabled research on plasticity-like effects in the human brain [12]. The **Paired Associative Stimulation (PAS)** protocol, based on the STDP protocol, synchronizes two different nervous system activators in time and neuronal pathway, and has been commonly applied to the primary motor cortex (M1). In 2000, Stefan et al. tested pathway excitability changes in the motor cortical output circuitry by pairing low-frequency peripheral stimulation of somatosensory afferents synchronously with TMS over M1, demonstrating reliable induction of motor cortical plasticity [13]. This innovative approach to TMS application opens the way to the possibility of inducing plasticity in humans in a non-invasive way, and several different protocols combining TMS pulses with other cortex activation stimuli have been developed [14–16].

Synchronization of cortex activation with the TMS pulse is needed to facilitate tract excitability; therefore, Thabit et al. (2010) activated the hand M1 cortex endogenously by asking participants to move their hand during a motor task [17]. Thus, the new task-related PAS protocol was designed by combining TMS cortical activation with a motor task, which required muscular movement 50 ms before the pulse. This PAS protocol potentiated corticospinal excitability and functional facilitation of task performance [17, 18]. In this way, they opened the door to inducing TMS-PAS plasticity in any cortical area that could be activated by a task and reachable by TMS, such as cognitive-related areas.

Recent TMS studies have reported improved cognitive processes, specifically memorization potentiation. These studies have mainly followed a repetitive TMS (rTMS) protocol (traditional or Theta Burst Stimulation) to induce functional enhancement in word-related memory [19–25], recognition of faces and buildings [20], and paired associative memory [26–29]. However, single-pulse TMS (spTMS) following a PAS paradigm that induces memorization capacity enhancement has not been previously demonstrated in humans.

Here, we introduce a new protocol derived from task-related PAS methodology to enhance memorization capacity in humans. We hypothesized that the combined stimulation of TMS and endogenous hippocampal activation would enhanced WM task performance. Moreover, for the first time, we demonstrated the viability of directing TMS to the hippocampus and neuromodulation to improve WM using a combined-stimuli protocol. These findings may have important implications in the use of NIBS to treat neurological diseases that are affected by memory impairment.

## Materials and methods

### Participants

96 healthy volunteers (age 19–43; 50 women) participated in this study. The participants were recruited during 2019–2021. None of the subjects had any past or present neurological disorders or pharmacological treatment on the day of the experiment. All participants provided written informed consent before the experimental intervention. The study was conducted in accordance with the Declaration of Helsinki and the Spanish National Research Council (CSIC) Ethics Committee approved all the procedures (ref 201850E062; 024/2019). At the beginning of the session, the subjects completed a questionnaire to collect demographic data such as **Age**, **Gender**, and **Visual Dominance**. Visual dominance was tested using the Point-a-Finger Test, where participants were instructed to point with their index finger to a 6-meter distant object and then, alternately close each eye to report with which eye had to do a greater correction [30]. In addition, participants were asked to look through an imaginary monocular microscope and to aim with an imaginary bow and arrow toward a distant object. The eye with the least correction in Point-a-Finger test, the one used to look into the monocular microscope and the one remained open when aiming, was considered to be the dominant eye.

Regarding the confidentiality of the collected data, each participant was assigned a unique code, along with their personal sociodemographic data, informed consent, and an opinion survey. These files remained in custody of the project administrator (corresponding author), while the number assigned to the subject was the label of the data that was later analyzed. Thus, there was no possibility of relating the records to the participants from whom they were obtained. The coded data were stored in our laboratory hard drives.

### Working memory task

**Fig 1:**
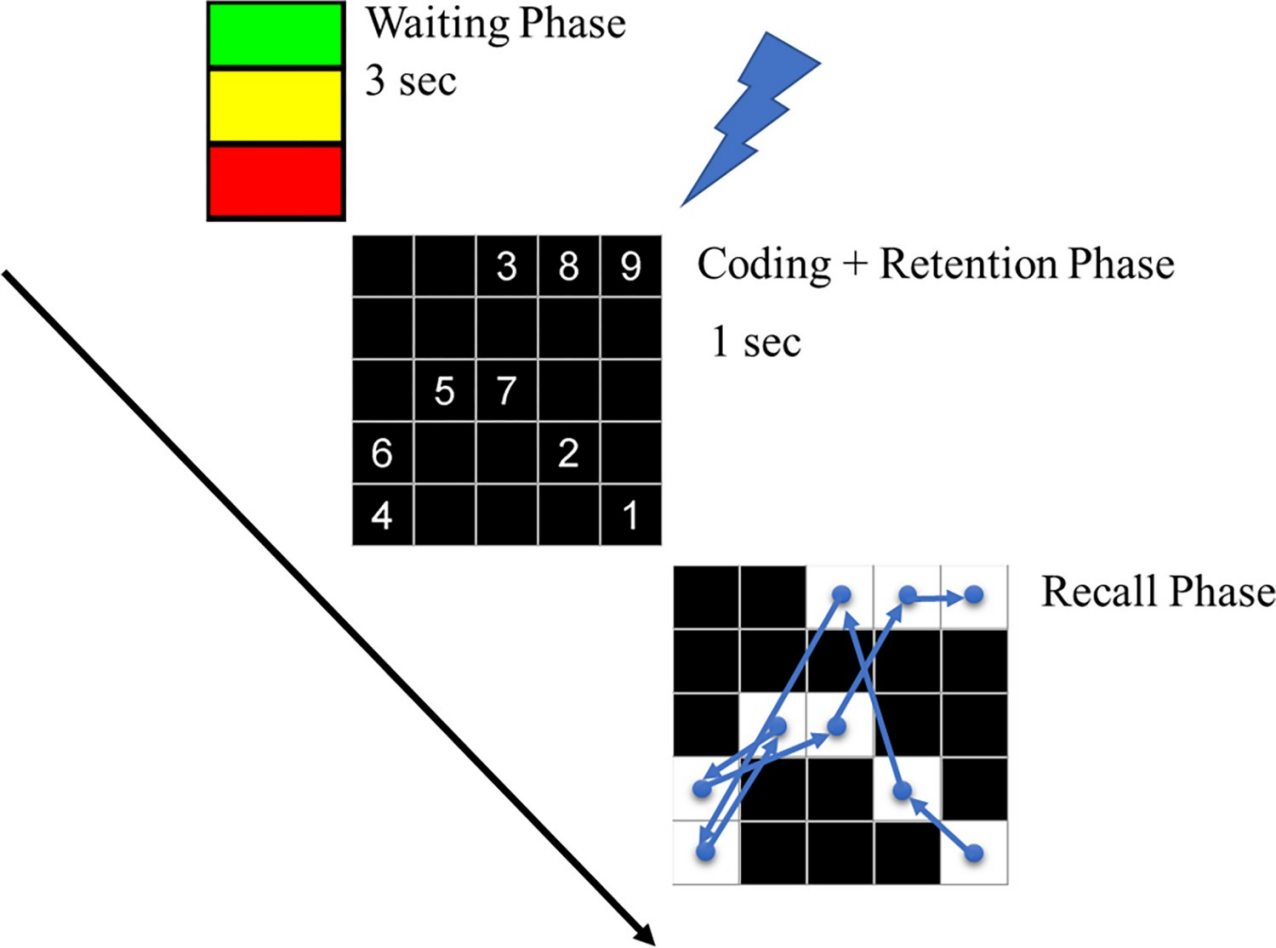
Working memory task. Representation of different phases of one WM task trial in temporal order: Waiting Phase: A traffic light visualization is displayed, indicating the upcoming trial initiation; Coding + Retention Phase: TMS is synchronized with the beginning of the phase; Recall Phase: The blue arrows represent a completely correct trial execution. The time duration of recall phase is dependent on the subject’s performance.

We adapted the “masking task” model (Fig 1) presented by Inoue and Matsuzawa, in 2007 [31]. We coded the program that ran the memorization task using MATLAB (MATLAB and Statistics Toolbox Release 2013b, The MathWorks, Inc., Natick, MA, United States).

The WM task was organized in three phases: 1) Waiting phase: a visual cue was presented, indicating the start of the memorization activity; 2) Coding and retention phase: nine numbers (one to nine) were presented in a random arrangement within a 5 x 5 matrix; 3) Recall phase: the numbers disappeared, leaving a white background as a clue, and the participant recalls the number arrangement by selecting the matrix squares following an incremental order. The number of successfully remembered items was recorded as the hit-items. The task is completed either when the subject makes a mistake or when the entire task is completed correctly. The hit-items of each trial (0–9) were registered using the same program running the WM task.

In order to synchronize an spTMS with the WM task in each trial, the task was composed of a coding and recall phase. In addition, in the recall phase, choices were not presented to rule out recognition and familiarity processes. This WM task was used to assess memorization capacity and complement the combined-stimuli intervention.

### Experimental design

**Fig 2:**
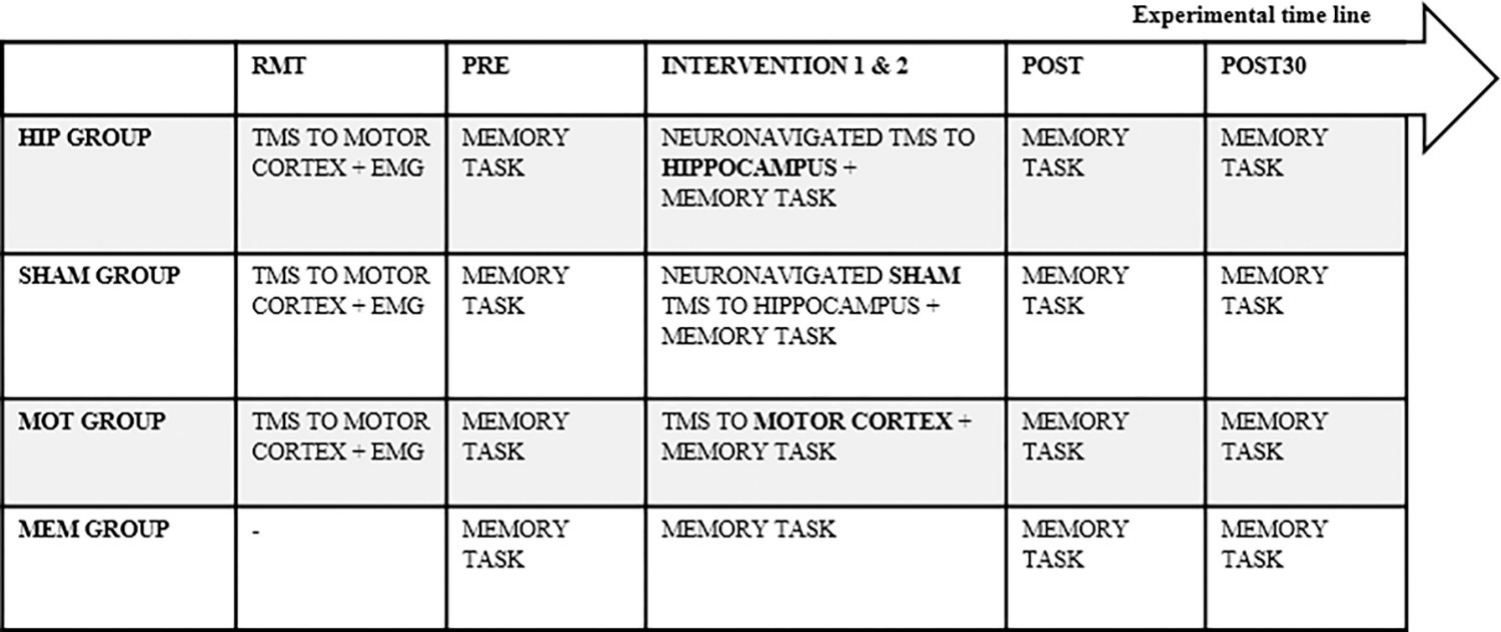
Experimental design timeline. Experimental structure representing the timeline of the experimental stages for the four different groups.

The present study was structured based on the comparison of the hippocampus experimental group (HIP) with the following three groups: sham stimulation group (SHAM), motor cortex group (MOT), and no stimulation/only memory task group (MEM). The four groups followed the same timeline (Fig 2).

1. **Resting Motor Threshold (RMT) assessment:** The experimental session began with the subjects seated in a chair and their dominant foot placed comfortably on a pillow wrapped in paper. The TMS hotspot in the M1 of Flexor Hallucis Brevis (FHB) muscle in the foot was found. We started with a TMS pulse of 50% of the maximum stimulator output and increased the intensity until we found the RMT. The RMT is the stimulation intensity that evokes five out of ten times the motor evoked potential (MEP) with a peak-to-peak amplitude of 50μV while the muscle is in a resting state [32]. This phase was not necessary for the MEM group. The RMT of the FHB muscle in the foot was assessed for methodological reasons (see section 2.4.2).
2. **Pre-assessment (PRE):** The subjects completed 25 trials of the WM task to evaluate their basal capacity of memorization (baseline score).
3. **Combined-stimuli intervention in two time-blocks (INT1 and INT2):** Neuronavigation calibration was completed in the HIP and SHAM groups, followed by subsequent pairing of spTMS with the WM task in the HIP, SHAM, and MOT groups. In the HIP and MOT groups, 120% of the FHB M1 RMT intensity was delivered in 25 task-pulse pairs in each intervention time-block (50 pulses in total). A frameless stereotactic Brainsight TMS Navigation system assisted with coil placement over the scalp of the subject to precisely direct magnetic stimulation to the hippocampus in the HIP and SHAM groups. Neuronavigation was based on an optical (infrared) position-sensor tracking method (NDI Polaris camera (Vicra/Spectra). We aligned the participant’s head and TMS coil position in 3D real space to the MNI152 image space on the screen. Then, we calibrated the central point of the coil with the coil handle trackers so that we could identify the TMS central point in the virtual space. Next, we designated 40 anatomical landmarks on the MRI image that we identified on the subject’s head based on three trackers placed on the subject’s head and using a tracked pointer to physically indicate the designated landmarks on the subject’s head and face. In this way, we could visualize the TMS coil center point location in relation to the head and brain structures and in real time from the physical space to the virtual space, enabling precise application of TMS to the hippocampus. TMS was applied to the contralateral hemisphere of visual dominance. This decision was based on the decussation of visual fibers (like the arrangement of motor fibers). The coil and stimulator utilized in both assessments and intervention time-blocks were a Double Cone Coil (DCC) and a Magstim 200^2^ stimulator from Magstim (Magstim, Whitland, United Kingdom). We used a DCC because it is able to reach deeper cortical targets [32]. The pulse waveform applied was monophasic.
4. **Post-assessment (POST):** The subjects performed 25 trials of the WM task immediately after the intervention, and their memorization capacity was evaluated.
5. **Post-30’-assessment (POST30):** 30 minutes after the intervention, the subjects performed 25 trials of the same WM task, and the memorization capacity was evaluated again.

#### HIP group

24 subjects (11 females) participated in the HIP group, which received the active combined-stimuli intervention, i.e. active TMS directed to the hippocampus in synchronization with WM task performance. Thus, the TMS is applied in the participants’ scalp corresponding to the temporal lobe.

Our TMS-task combined intervention was based on the convergence of **two stimuli** in the same neuronal population. In this study, one stimulus was a spTMS to the hippocampal formation in the medial temporal lobe, and the other stimulus was endogenous activation of the hippocampus due to WM task performance. In the case of cognitive tasks, specifically in the case of the hippocampus, it is difficult to determine the exact time at which the hippocampus is activated. This is due to the complexity of cognitive processes and the location of the hippocampus in the brain, which its particular activity is difficult to record using scalp electroencephalography (EEG) or other non-invasive techniques. The lack of well-known activation time during a cognitive task makes it difficult to synchronize this activation with a TMS stimulus. Thus, we combined exogenous spTMS with endogenous hippocampal activity by synchronizing spTMS with the coding phase of the WM task and compared the designed intervention group with three parallel groups.

Thus, we applied spTMS synchronized with the beginning of the encoding phase of the memorization task when the items to memorize were presented (see description in the *Working Memory Task* section). The paired task-pulse frequency depended on the performance time of each subject in each trial.

#### SHAM group

24 subjects (12 females) participated in the SHAM experimental group. The experimental procedures during RMT measurement and neuronavigation calibration were the same as those in the active HIP group; however, during the sham intervention, a DCC TMS was placed on the subject’s scalp directed at the hippocampus without the administration of the TMS pulse. The sound of the stimulation was simulated using another coil placed perpendicular to the participant’s scalp. The subjects were blinded to the condition of the TMS they received (active or sham). We conducted this comparative group to rule out placebo-type processes and verify that the changes in memorization capacity were due to the active stimulation of the combined-stimuli intervention.

#### MOT group

24 subjects (15 females) participated in the MOT group. In the intervention time-blocks, the subjects performed the WM task combined with spTMS directed to their FHB M1. Thus, the TMS is applied in the scalp corresponding to the frontal lobe. We chose not to guide the TMS by neuronavigation and, instead, we rely on the individual hot-spot by a motor mapping assessment. We designed this comparative group to address the brain localization factor of the combined-stimuli intervention.

#### MEM group

24 subjects (12 females) participated in the MEM group. In the intervention time-blocks, the subjects performed the WM task without receiving or applying neuronavigation, RMT, and TMS. We designed this parallel group to assess the basal capacity for memorization restricted to task training.

### Hippocampal stimulation feasibility calculations and statistical analysis

The statistical procedures were calculated with the aid of statistical software IBM SPSS (IBM Corp. Released 2010. IBM SPSS Statistics for Windows, Version 19.0. Armonk, NY, United States) and a *p* value < 0.05 was considered significant for every statistical analysis.

#### Population sample homogeneity analysis

*Group homogeneity*. Applying Pearson’s chi-squared test (χ2 test), we ruled out the existence of unbalanced weighting of different characteristics (**Age**, **Gender**, **Visual Dominance**, and **RMT**) among the 4 groups (HIP, SHAM, MOT, and MEM) that could bias the result of the effects of memory-related combined-stimuli intervention.

*Baseline score homogeneity*. Due to the significant weight as a covariate factor and its interaction with Group and Time variables, we statistically analysed Baseline score factor in combined-stimuli intervention effects. We used three levels for Baseline score factor: Low baseline score (LB), Medium baseline score (MB), and High baseline score (HB). Within each group (HIP, SHAM, MOT, and MEM), the subjects were distributed considering their baseline hit-items score as follows: participants with the baseline score within the lower 33% of the group were classified as LB, within the middle 33% were classified as MD, and within the upper 33% were classified as HB.

Calculating Pearson’s chi-squared test (χ2 test), we assessed the distribution of the population’s characteristics between the different baseline categories (Low, Medium, and High) and possible bias in the memory-related intervention effects. The variables analysed between the three categories were the same as previously considered: **Age**, **Gender**, **Visual Dominance**, and **RMT**.

#### Human hippocampus depth measurement and comparison in MRI

Stimulation of the hippocampal area does not trigger an assessable evoked potential; therefore, it is not possible to directly establish the required intensity of spTMS to reach the target. Thus, we used an indirect measure. We compared the depth of the hippocampus with the deepest M1 of the frontal lobe (foot M1) and estimated the capacity to stimulate the hippocampus based on TMS parameters that elicit MEPs in foot M1.

**Fig 3:**
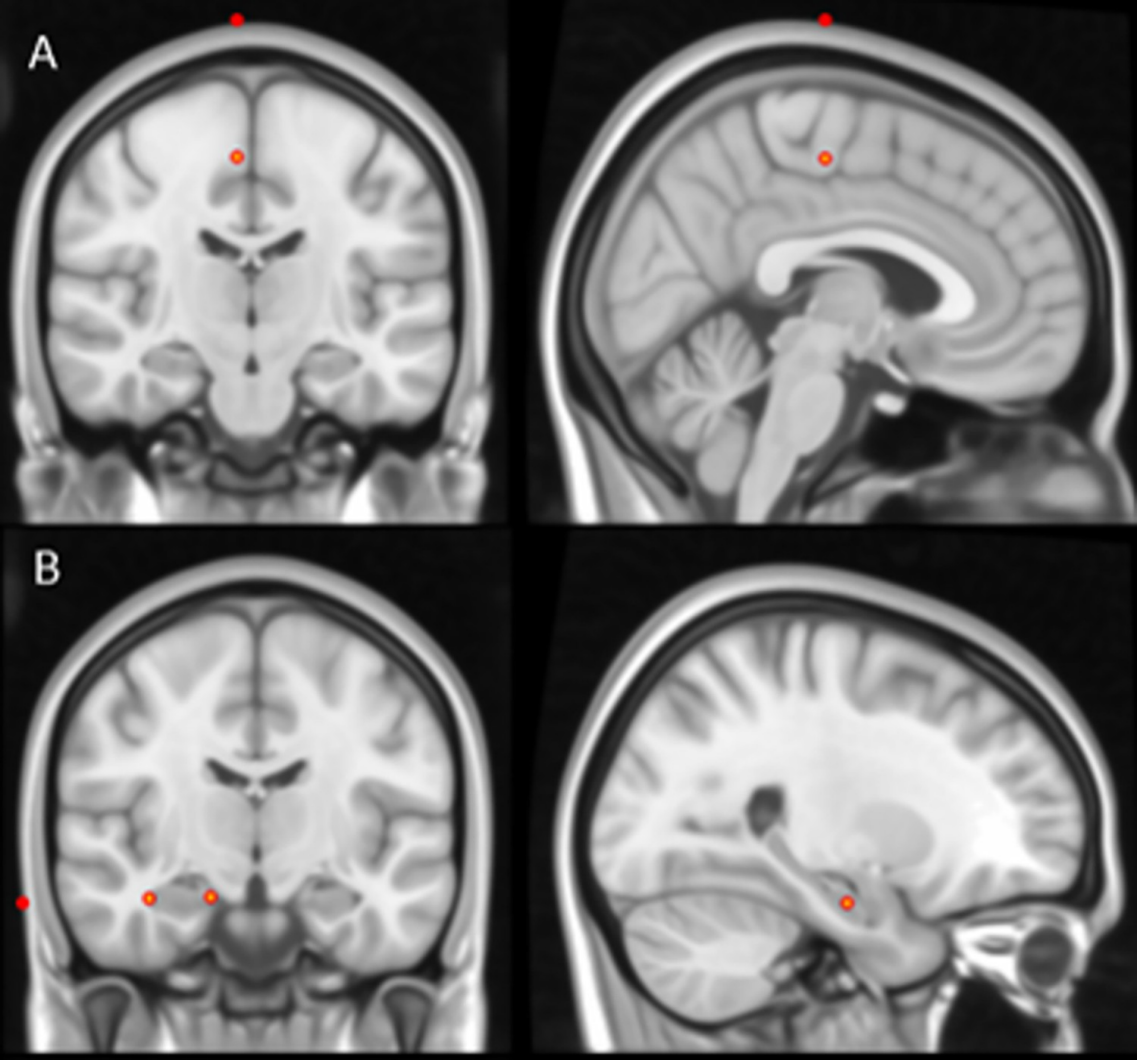
Key locations represented in coronal (left) and sagittal (right) view in MRI. A. Primary foot motor cortex location and the corresponding frontal surface location. B. Most distal and proximal points of the hippocampus and corresponding temporal surface location.

Anonymous single-subject MRI images of the entire brain of eight healthy subjects were analyzed in this study. With the aid of MRIcro software, the coordinates of the following key locations were registered in each subject’s MRI: deepest foot cortex [33], respective frontal surface, most distal and proximal points of both hippocampi and respective temporal surface (Fig 3A and 3B).

Each key location was identified in the right and left hemispheres. The hippocampal boundaries were defined to include the CA1 through CA4 areas of the hippocampus-proper, dentate gyrus, and subiculum in the hippocampal head in the foot M1 coronal plane. Foot cortex locations were selected based on functional MRI (fMRI) studies [34] and the Haines Neuroanatomy Atlas [35]. The mean lateromedial hippocampal length, depth of the hippocampi at its proximal, middle and distal points, and foot cortex depth were calculated. The depths of the middle and lateral average points of the hippocampi were compared with the depth of foot M1 in individual MRIs scans.

#### Learning characterization of WM task

The index that we used to measure learning throughout the experimental session was the **memorization capacity** measured by the proportion of hit-items of each time-block (INT1, INT2, POST, and POST30) in comparison with hit-items in the baseline-block (PRE). The performance data of the MEM group subjects were statistically analyzed by a repeated measures ANOVA of 1-factor, TIME, with four levels, one for each time-block. Through this analysis, we were able to verify the percentage of learning that occurred solely because of WM task performance.

#### Combined-stimuli intervention effects on memorization capacity

The successfully remembered items (hit-items) in the WM task trials rated the memorization capacity. We calculated the mean hit-items of the INT1, INT2, POST and POST30 time-blocks and their proportion related to the mean hit-items of PRE. When sphericity was not assumed, we used the Greenhouse-Geisser correction.

We found that the memorization capacity improvements of the LB, MB, and HB subjects were different. Thus, we included the **Baseline score** variable (Baseline raw data) as a covariable factor in a mixed ANOVA with the within-subjects factor **Time** (INT1, INT2, POST and POST30) and between-subjects factor **Group** (HIP, MOT, SHAM, and MEM).

Due to the significant weight of the Baseline score factor as a covariate, we performed a mixed ANOVA with the within-subjects factor **Time** (INT1, INT2, POST and POST30), between-subjects factor **Group** (HIP, MOT, SHAM, and MEM), and within-subjects factor **Baseline score** (LB, MB, and HB). We then analyzed the effects of combined-stimuli intervention for each Baseline score level with three separate follow-up ANOVAs (LB, MB, and HB) with the within-subjects factor **Time** (INT1, INT2, POST, and POST30) and between-subjects factor **Group** (HIP, MOT, SHAM, and MEM).

For each ANOVA, Bonferroni pairwise multiple comparison post-hoc tests were performed.

## Results

We assessed combined-stimuli intervention effects on the memorization capacity 1) ruling out population bias, 2) measuring and comparing hippocampus depth, 3) characterizing WM task associated learning and 4) comparing the memorization proportion between groups, WM task time-blocks and baseline scores.

### Population sample analysis results

The comparison of the Group variable of four levels (HIP, MOT, SHAM, and MEM) with 96 subjects’ independent variables **Age** (χ2(60) = 64.067, p = 0.336), **Gender** (χ2(3) = 1.503, p = 0.682), **Visual Dominance** (χ2(3) = 3.000, p = 0.392) and **RMT** (χ2(44) = 41.386, p = 0.584), revealed that there is not any statistically significant association between these factors and the distribution of the population between groups. Thus, there is not any bias regarding these factors in the group’s population (Table 1).

**Table 1:** Analyzed demographic data.

		Age	Subjects	Visual Dominance		RMT	Memory Baseline
		Mean Yrs. ± SD	No.	Right	Left	Mean % ± SD	Mean hit-items ± SD
**HIP group**	LB	26 ± 3	8 (2 f)	8	0	43 ± 8	3.04 ± 0.37
	MB	25 ± 2	8 (3 f)	7	1	41 ± 10	3.91 ±0.19
	HB	27 ± 5	8 (6 f)	7	1	42 ± 6	5.24 ± 0.72
**SHAM group**	LB	22 ± 2	8 (6 f)	7	1	47 ± 4	3.34 ± 0.38
	MB	26 ± 5	8 (3 f)	6	2	42 ± 6	4.19 ± 0.19
	HB	25 ± 6	8 (3 f)	8	0	45 ± 7	5.26 ± 0.62
**MOT group**	LB	23 ± 3	8 (6 f)	6	2	41 ± 8	3.23 ± 0.25
	MB	27 ± 6	8 (7 f)	7	1	43 ± 6	4.21 ± 0.39
	HB	23 ± 2	8 (2 f)	5	3	41 ± 7	5.29 ± 0.31
**MEM group**	LB	30 ± 6	8 (2 f)	5	3	.	3.54 ± 0.27
	MB	26 ± 7	8 (4 f)	7	1	.	4.39 ± 0.23
	HB	28 ± 6	8 (6 f)	7	1	.	5.56 ± 0.60

Age, number of subjects, visual dominance, and memorized hit items at baseline categorized by experimental group (HIP: Hippocampus group; SHAM; Sham group; MOT: Motor Cortex group; MEM: Memorization task group) and memory capacity baselines (LB: Low baseline subjects; MB: Medium Baseline subjects; HB: High Baseline Subjects).

Baseline score variable of three levels (Low, Medium, High) assessment for the 96 subjects regarding the distribution of the independent variables **Age** (χ2(40) = 42.871, p = 0.349), **Gender** (χ2(2) = 0.584, p = 0.747), **Visual Dominance** (χ2(2) = 0.600, p = 0.741) and **RMT** (χ2(44) = 42.800, p = 0.523), revealed that there is not any statistically significant association between these factors and the distribution of the population between baseline scores. Thus, there is not any existing bias regarding these factors in the different baseline group’s population.

### Human hippocampus depth measurement and foot M1 comparison

On the one hand, the midpoint at a coronal view of the right and the left hippocampi for individual MRIs were at a mean ± SD depth of 49.25 ± 3.68 mm and 51.91 ± 7.23 mm respectively. On the other hand, the depth of foot M1 in right and left hemispheres for individual MRIs were at 43.24 ± 1.48 mm and 44.99 ± 1.68 mm respectively. The difference between hippocampal middle point and foot M1 depths is 6.01 ± 3.7 mm for the right hemisphere and 6.92 ± 4.83 mm for the left hemisphere.

Although there is a difference between foot M1 and the middle point of hippocampus of 6,47 mm (being hippocampus deeper), the width of the human hippocampus was on average 19.84 ± 2.85 mm (in accordance with Burggren and colleagues (2008) [36]). This means that the hippocampus extends from its middle point to a minimum of 8.5 mm on either side, which implies that the depth of the foot M1 falls within the range of the hippocampus. In this way, we estimate that the intensity that triggers the foot M1 activation will also activate the hippocampal nervous cells when applying the TMS pulse towards that location.

### Learning characterization of WM task

We characterized the extent of learning that an individual can develop solely due to its repetition (MEM group data exclusively).

**Fig 4:**
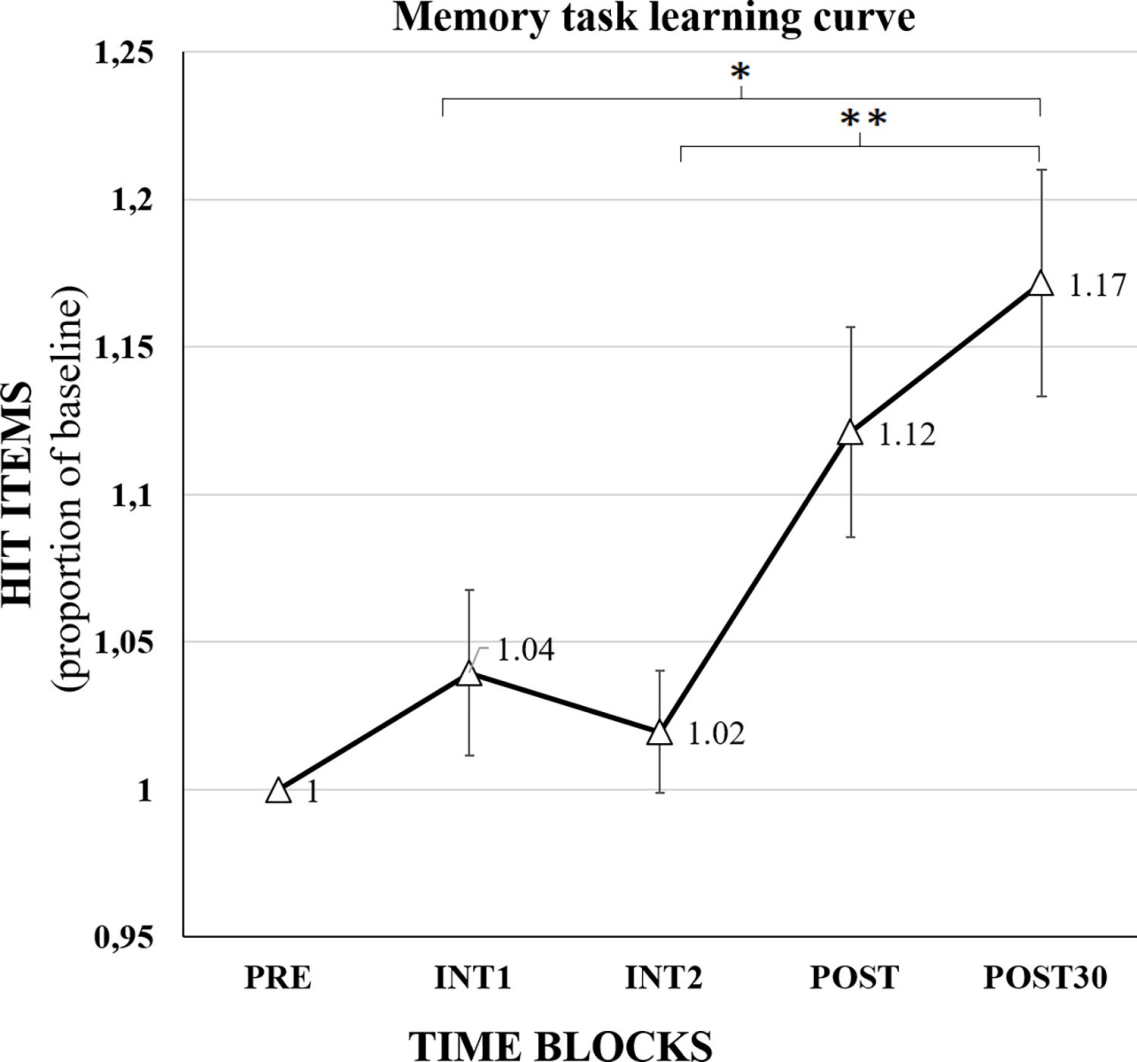
Memorization capacity of the MEM group. Mean and standard error of hit-item proportion in each time-block compared to the baseline score showing a learning curve derived from the training without active or sham stimulation of the WM task used in this combined-stimuli study (MEM group). * for p<0.05 and ** for p<0.01.

The ANOVA of 1-factor, TIME, of four levels of times resulted in a significant main effect of Time (F (3, 95) = 5.085, p = 0.003). The post-hoc pair comparison with Bonferroni correction revealed that the POST30 (Mean = 1.172 SD = 0.188) was significantly higher than the INT1 (Mean = 1.040 SD = 0.138, p = 0.023) and INT2 (Mean = 1.020 SD = 0.101, p = 0.006) time-blocks (Fig 4). The results indicated that the practice of this task by itself triggers memorization mechanisms that in the long term makes an enhancement on WM compared to the first half of the experimental session. The long-term memorization in POST30 is on average 17.2% higher than the baseline PRE time-block with a standard error of 0.04.

In addition, we demonstrated that it was feasible to play the WM task and no subjects had any complaints about gameplay, comfort or performance. The task that we programmed and involved in the combined-stimuli intervention was functional and demonstrated a learning curve that contextualize the further results.

### Combined-stimuli intervention effects on WM task

Regarding the mixed ANOVA with the intra-subject factor **Time** (INT1, INT2, POST and POST30), inter-subject factor **Group** (HIP, SHAM, MOT and MEM) and the covariable **Baseline score**, a significant main effect on **Time** (F(2.765, 251.651) = 5.968, p = 0.001) and **Baseline Score** (F(1, 91) = 54.145, p = 0.000) was found.

The omnibus mixed ANOVA with the intra-subject factor **Time** (INT1, INT2, POST and POST30), inter-subject factor **Group** (HIP, MOT, SHAM, and MEM) and **Baseline score** (Low, Medium and High) revealed a significant main effect on **Time** (F(3, 252) = 27.438, p = .000), **Group** (F(3,84) = 3.069, p = 0.032) and **Baseline Score** (F(2, 84) = 27.075, p = .000); and a significant interaction effect of **Group X Baseline Score** (F(6,84) = 3.282, p = 0.006).

**Fig 5:**
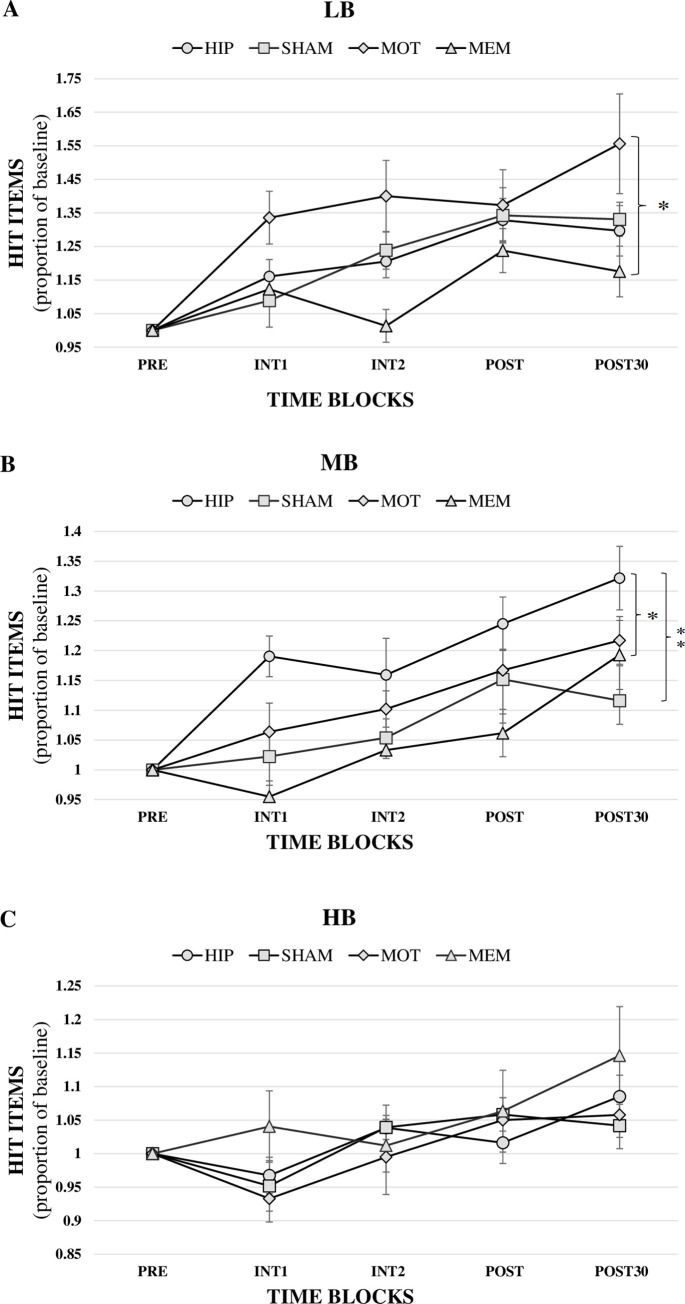
Memorization capacity compared between groups at different time-blocks. Mean and standard error of hit-item proportion in each time-block compared to the baseline score. A. Low baseline score subjects, B. Medium baseline score subjects, C. High baseline score subjects. * for p<0.05 and ** for p<0.01 of Group interaction post-hoc statistics reported in Results section in follow-up ANOVAs.

As the interaction Group X Baseline Score was significant, we performed 3 separate follow-up ANOVA (one for each Baseline score). We analyzed the different Baseline score levels separately in three mixed ANOVAs with the intra-subject factor **Time** (INT1, INT2, POST and POST30) and inter-subject factor **Group** (HIP, SHAM, MOT, MEM) (Fig 5).

In **LB** subjects mixed ANOVA, significant main effects on **Time** (F(3, 84) = 9.041, p = 0.000) and **Group** (F(3,28) = 3.236, p = 0.037) were found. The post-hoc pairwise comparison with Bonferroni correction revealed that the MOT group (Mean = 1.416 SD = 0.064) had a significantly higher memorization capacity than the MEM group (Mean = 1.138 SD = 0.064, p = 0.027).(Fig 5A).

In **MB** subjects mixed ANOVA, significant main effects on **Time** (F(3, 84) = 11.349, p = 0.000) and **Group** (F(3,28) = 6.120, p = 0.002) were found. The post-hoc pairwise comparison with Bonferroni correction revealed that the HIP group (Mean = 1.229 SD = 0.030) had a significantly higher memorization capacity than SHAM Group (Mean = 1.086 SD = 0.030, p = 0.013) and MEM Group (Mean = 1.061 SD = 0.030, p = .003) (Fig 5B).

Finally, in **HB** subjects mixed ANOVA, a significant main effect on **Time** (F(3, 84) = 9.335, p = .000) was found, but significant main effects on the Group factor were not found (p = 0.640) (Fig 5C).

Regarding LB subjects, the MOT intervention increased 42% their memorization capacity on average, while the SHAM group improved 25% and the MEM group improved 14%. Regarding MB subjects, HIP intervention achieved an average improvement of 23%, while the SHAM and the MEM interventions remained at 9% and 6%, respectively. Therefore, we conclude that this stimulation protocol is dependent on the interaction between stimulation location and the subjects’ basal memorization capacity.

## Discussion

This study presents the design and research of an innovative methodology based on the combined application of spTMS and endogenous hippocampal activity during the coding phase of a WM task. The present results show that the application of this new protocol significantly improves memorization capacity.

The WM task, utilized as part of the combined protocol, was characterized without interactions (TMS or Sham) to define the average capacity to memorize items in a single session. The outcome score of the MEM group increased significantly in the POST30 time-block in comparison with the PRE time-block. Nevertheless, the MOT intervention had a significant potentiating effect that improved the ability to memorize and perform the WM task in LB subjects, and the HIP intervention had a significant potentiating effect on MB subjects.

LB subjects showed greater improvement even without active stimulation compared to MB and HB subjects. Even so, LB subjects in the MOT group, with an improvement of 42%, performed the memorization task 1.66 times better than LB subjects in the SHAM group (25% of improvement) and 3 times better than LB subjects in the MEM group (14% of improvement). MB subjects of the HIP group (23%) improved their memorization capacity by 2.5 times compared to the SHAM group (9%) and 3.75 times compared to the MEM group (6%). In HB subjects, neither the HIP nor the MOT intervention induced changes in the memorization capacity of the subjects compared to the SHAM or MEM groups. Thus, we conclude that at baseline, HB subjects performed close to their maximum capacity, and a ceiling effect was likely to occur because neither training (SHAM and MEM) nor combined-stimuli facilitation (HIP and MOT) improved memorization in HB subjects.

The idea that brain resources supporting Short-Term Memory (STM) or WM are limited is not new [37], and has been the subject of significant research efforts in the past [38]. We know that WM is limited by a retention capacity of around four items [39], although this capacity is dependent on the nature and number of items in visual STM [40].

In our study, memorization involved nine visuospatial items. In the MEM group at POST30, the mean retention capacity ranged between 3.04 items (a LB subject) and 7.84 items (a HB subject). The average retention capacity in all groups of HB subjects was 5.34 items at PRE and 5.78 items at POST30, while LB subjects in the MOT group memorized on average 3.23 items at PRE and 5 items at POST30, and MB subjects in the HIP group memorized on average 3.91 items at PRE and 5.15 items at POST30. Seven subjects memorized a mean of 7 or more items at POST30; six of these participants were HB subjects and one was an LB subject in the MOT group who started with a memorization capacity of 3.08 items. Based on these data, we characterized the intrasession memorization capacity of this WM task as 5±2 items.

Given that differences between groups in HB subjects were not statistically significant and due to the limited capacity of WM neural function, we conclude that this combined-stimuli protocol promotes an improvement in those resources that are not fully potentiated in LB and MB subjects. Overall, we deduce that this combined protocol enhances the neuronal pathway responsible for memorization no more than the maximum functionality of the subjects’ neural capacity. However, further research is needed to demonstrate cortical plasticity induction by this combined-stimuli intervention.

### Location and baseline score interaction: Physiology of memory

As previously mentioned, LB subjects benefited from the combined WM task with spTMS over M1, while MB subjects benefited from the combined WM task with spTMS over the hippocampus. This finding revealed that these two cortical areas are involved in numerical and visuospatial WM task performance. We hypothesize that the hierarchy of cognitive processes in WM determines the brain location where combined-stimuli intervention will be effective.

Completing a WM masking-task requires encoding and retaining the associations between the presented items and correct matrix locations. Thus, we can infer that this task utilizes WM based on the coding, storage and manipulation processes involved [41]. Specifically, the information in this task was visual, numerical, and spatially sequenced.

The motor cortex is associated with sequence order coding. Carpenter et al. observed activity in the motor cortex of monkeys during sequential association. They reported different profiles of cellular responses within the motor cortex that responded to specific temporal and spatial positions as well as the interaction of these two variables [42, 43]. Furthermore, previous studies have indicated that hippocampal activation is critical for WM when subjects remember object-location associations to identify items in space [7, 10, 44].

To perform the presented WM task, individuals require at least two cognitive processes: first, coding items and their order, presumably relying on the motor cortex, and second, retaining the associated information between spatial locations, presumably relying on the hippocampus.

We hypothesize that in LB participants, the neural pathway encoding the numbers and their order is mainly activated in M1 and is less efficient than in MB and HB subjects. Pairing TMS repeatedly with active neuronal cells in the M1 may induce plastic mechanisms, which would enhance M1 function to encode and process items. HIP intervention would not facilitate memorization because of lower spatial information retention and hippocampal activity.

Conversely, the MB participants’ coding process may be functional, while the retention of spatial information is not significantly strengthened. In this case, HIP intervention would enhance the hippocampal neural ensembles in charge of retaining spatial information. Through the strengthening of synapses, the retention and manipulation of information capacity can be improved. Then, the MOT intervention would not be significantly effective because these subjects already encoded items and their order efficiently.

However, we have to consider that the LB group is significantly different from the MEM group and, despite having an improvement compared to the SHAM and HIP groups, the difference is not statistically significant. The fact that there is no significant difference could suggest that there are other variables in these groups but not in the MEM group (such as the sound of the TMS) that could be affecting the results. Due to the significance of the baseline variable in the effect of combined stimulation, the sample that is divided into groups and subgroups is ultimately small and therefore, to clarify these secondary variabilities, this protocol should be tested in a larger sample in the future.

Nevertheless, further research is needed to investigate the plastic changes that may involve WM task outcome changes found in this study.

### Methodological considerations: Combined-stimuli design characteristics

#### Foot motor cortex and hippocampus depth comparison

The proposed methodology is novel in orienting TMS to the hippocampus to combine hippocampal endogenous activity with spTMS. The comparison between foot M1 and the hippocampus determined that the depth of both brain regions is comparable, and that the technique is applicable to both hemispheres. Moreover, we designed a combined-stimuli protocol to apply an intensity equivalent to 120% of the RMT of the foot M1. With these parameters, it is highly probable that TMS activates the hippocampal nervous cells located at a depth equivalent to the M1 foot representation. Furthermore, the foot M1 and hippocampus have comparable cytoarchitecture: both areas are populated by pyramidal neurons and part of their arrangement is parallel to the contiguous scalp, following a columnar distribution along the cortical gyrus [45, 46].

#### TMS-task synchronization

To select the synchronization time between the spTMS and the phase of the task, we relied on Hebbian principles that define the change in synaptic force as a function of co-activation (Hebb, 1949). When the items were presented to the subjects, they had to memorize them in order and space; thus, we assumed that the application of a TMS pulse at that time would be paired with the endogenous activation of the neurons responsible for that codification and retention. To confirm or refuse this hypothesis, further research with respect to the optimal Inter Stimulus Interval (ISI) between TMS and endogenous activity during WM tasks is needed.

The difference between the presented combined-stimuli methodology that pairs TMS (an exogenous stimulus) with hippocampal activity when performing a memory task (endogenous stimulus), with the original PAS methodology is the nature of the second stimulus. The memory task that evokes activity in the hippocampus provides a way to associate its activity to TMS, which otherwise would not be possible. However, only when the effect of the ISI factor will be researched, could be confirmed that the repeated combination of activities (exogenous and endogenous) induces potentiation by STDP induction. If this was not the case, then the mechanisms induced by this combined-stimulation should be investigated. In addition, our results showed that this combination is effective depending on the basal level of memorization capacity. As previously discussed, the baseline score reflects the level of functionality of the subject’s neuronal pathways. Hence, the synaptic strength of the neuronal pathway at a specific level of functionality would be sensitive to this combined-stimuli intervention and would dictate which areas would be the best targets for TMS synchronization.

### Future considerations and study limitations

The participants in this study were between 19 and 43 years of age, which can be considered a population of young adults. We propose that future studies of this new combined-stimuli protocol for the improvement of memorization seek the feasibility of its application in elderly or Mild Cognitive Impairment (MCI) populations. WM is a fundamental ability for daily living and tends to decline significantly with age [47]. The adaptation of a combined-stimuli intervention to an elderly population and outlining its memory potentiation efficacy could lead to a potential therapeutic approach to alleviate memory decline or other related cognitive capacity conditions.

The limitations of this combined-stimuli intervention study are the following. First, direct probing of TMS-triggered activation at the hippocampal level is not yet possible due to the lack of directly measurable TMS effects, such as MEPs for M1. Although we assumed that using a TMS intensity level capable of stimulating the foot M1 would stimulate the hippocampus (at least the most proximal portion), TMS stimulation in deeper locations may be more diffuse. Therefore, further research on TMS activation of the hippocampus and surrounding areas in the temporal lobe is necessary to ascertain whether specific hippocampal activation or stimulation of other temporal lobe areas leads to memory potentiation.

Second, the effect of ISI on TMS and endogenous memory activity in our design has not been systematically analyzed. In our experiments, the beginning of the coding phase in the WM task and the TMS pulse were synchronized. Unveiling whether our results on WM task outcome facilitation correspond to a Hebbian mechanism, and therefore likely triggering STDP-like plasticity, would require a systematic investigation of the ISI variable.

Regarding methodological limitations, although the hippocampal-oriented stimulation in the HIP group has been supported by neuronavigation, the MRI of the 96 subjects who participated in the study were not available. Thus, as explained in Material and methods section, the neuronavigation in HIP group was conducted with the aim of a universal brain map of 152 MRIs (MNI152). In the future, this stimulation protocol could be improved with neuronavigation with individual MRIs to avoid variability. Although the hippocampus is a relatively large structure, it is also deep, which makes its stimulation less precise. Therefore, it is necessary to use all possible techniques or adjustments to ensure that stimulation is as precise as possible.

## Conclusion

In this study, we present a new combined-stimuli protocol in which TMS is focused on hippocampal-oriented stimulation and paired with a WM task that triggers endogenous hippocampal activity, resulting in increased intrasession memorization capacity. In addition, we found that these memory effects were dependent on the stimulation location and the subject’s basal memorization capacity. Future research is required to measure hippocampal feedback and assess the effects of different ISIs to unveil the changes in neural mechanisms that enhance the ability to memorize. Finally, this combined-stimuli protocol is potentially significant for individuals affected by memory decline.
